# Guías de práctica clínica: estudio cualitativo sobre su implementación en el sistema de salud de Chile

**DOI:** 10.26633/RPSP.2017.67

**Published:** 2017-04-28

**Authors:** Paloma Herrera, Valentina Fajreldin, María Francisca Rodríguez, Patricia Kraemer, Carolina Mendoza, Ignacio Pineda, Pamela Burdiles, Marco Cornejo, Julio Villanueva, María Dolores Tohá, Alonso Carrasco-Labra

**Affiliations:** 1 Departamento Secretaría Técnica de Garantías Explícitas en Salud (GES) y de Coordinación Evidencial y Metodológica, Subsecretaría de Salud Pública Departamento Secretaría Técnica de Garantías Explícitas en Salud (GES) y de Coordinación Evidencial y Metodológica, Subsecretaría de Salud Pública, Ministerio de Salud Chile Departamento Secretaría Técnica de Garantías Explícitas en Salud (GES) y de Coordinación Evidencial y Metodológica, Subsecretaría de Salud Pública, Ministerio de Salud, Chile.; 2 Unidad de Odontología Basada en Evidencia e Instituto de Investigación en Ciencias Odontológicas Facultad de Odontología Chile Unidad de Odontología Basada en Evidencia e Instituto de Investigación en Ciencias Odontológicas, Facultad de Odontología, Universidad de Chile, Chile.; 3 Facultad de Medicina Universidad San Sebastián Santiago Chile Facultad de Medicina, Universidad San Sebastián, Santiago, Chile.

**Keywords:** Guías de práctica clínica como asunto, sistemas de salud, Chile, Practice guidelines as topic, health systems, Chile, Guias de prática clínica como assunto, sistemas de saúde, Chile

## Abstract

**Objetivo.:**

Caracterizar el proceso de implementación, barreras y facilitadores de recomendaciones basadas en evidencia en el contexto del desarrollo de guías prácticas clínicas (GPC) generadas por el Ministerio de Salud de Chile, a fin de brindar propuestas para la optimización del proceso.

**Métodos.:**

*Estudio cualitativo del tipo “investigación-acción”. Se realizaron 19 entrevistas semiestructuradas y se armaron nueve grupos de discusión a distintos niveles del sistema público de salud chileno. El análisis se realizó mediante el* software *Atlas ti® y en forma manual, desde un marco de análisis de contenido, mediante la categorización y codificación de la información según dimensiones preespecificadas y con la inclusión de categorías emergentes cuando fue pertinente.*

**Resultados.:**

El principal desafío de implementación de recomendaciones mencionado es la falta de un proceso explícito y estructurado. Los actores del sistema de salud reconocen dificultades dependientes del contexto al momento de usar las recomendaciones. En esta experiencia inédita de revisión institucional, los participantes sugirieron una serie de estrategias a poner en práctica para superar dichos desafíos, representadas en un flujograma de gestión optimizada para el desarrollo e implementación de GPC. El mismo proceso ha permitido tomar conciencia de la importancia de la implementación de GPC en Chile.

**Conclusión.:**

Tras caracterizar el proceso de implementación, barreras y facilitadores se articuló un plan de implementación de recomendaciones que permitiría orientar y monitorizar dicho proceso. Hacer partícipes del proceso de revisión a informantes claves dentro y fuera del Ministerio de Salud facilitaría la implementación de estrategias y la introducción de mejoras al proceso de desarrollo de GPC. Estudios de este tipo deberían ser realizados en médicos y pacientes para complementar la información recogida.

## Abstract (en)

**Objective.:**

Characterize the implementation process, barriers, and facilitators of evidence-based recommendations in the context of developing clinical practice guidelines (CPGs) generated by the Ministry of Health of Chile, in order to make proposals to optimize the process.

**Methods.:**

Qualitative “action-oriented research” study. Nineteen semi-structured interviews were conducted and nine discussion groups were organized at various levels of the Chilean public health system. The analysis was conducted using Atlas.ti® software and manually, in a content analysis framework, by categorizing and coding information according to pre-specified dimensions and with the inclusion of emerging categories where relevant.

**Results.:**

The main challenge mentioned with regard to implementing recommendations is the lack of an explicit and structured process. Actors in the health system recognize difficulties specific to the context in which the recommendations are followed. In this unprecedented institutional review, participants suggested a series of strategies that could be implemented to overcome these challenges, presented in a management flow chart optimized for the development and implementation of CPGs. This process has raised awareness of the importance of implementing CPGs in Chile.

**Conclusion.:**

After characterizing the implementation process, barriers, and facilitators, a plan to implement recommendations was developed in order to guide and monitor the process. It would facilitate the implementation of strategies and the introduction of improvements to the CPG development process if key informants inside and outside of the Ministry of Health were included in the review process. Studies of this kind should be conducted with physicians and patients in order to complement the collected information.

## RESUMEN

**Objetivo.:**

Caracterizar o processo de implementação, as barreiras e os fatores facilitadores de recomendações baseadas em evidências no contexto do desenvolvimento de guias de prática clínica (GPCs) criados pelo Ministério da Saúde do Chile, a fim de fazer propostas para a otimização do processo.

**Métodos.:**

Estudo qualitativo do tipo “investigação-ação”. Foram realizadas 19 entrevistas semiestruturadas e organizados nove grupos de discussão em diferentes níveis do sistema público de saúde chileno. A análise foi realizada com o software Atlas ti® e de forma manual utilizando um quadro de análise de conteúdo, mediante a categorização e codificação das informações segundo dimensões pré-especificadas e com a inclusão de categorias emergentes, quando pertinente.

**Resultados.:**

O principal desafio identificado para a implementação das recomendações foi a falta de um processo explícito e estruturado. Os atores do sistema de saúde reconhecem dificuldades dependentes do contexto no momento de usar as recomendações. Nesta experiência inédita de revisão institucional, os participantes sugeriram uma série de estratégias que podem ser postas em prática para superar tais desafios, representadas em um fluxograma de gestão otimizada para o desenvolvimento e a implementação dos GPCs. O mesmo processo tem gerado conscientização sobre a importância da implementação de GPCs no Chile.

**Conclusão.:**

Uma vez caracterizado o processo de implementação, as barreiras e os fatores facilitadores, articulou-se um plano de implementação de recomendações que permitirá orientar e monitorar esse processo. O envolvimento de informantes-chave dentro e fora do Ministério da Saúde no processo de revisão poderá facilitar a implementação de estratégias e a introdução de melhorias no processo de desenvolvimento de GPCs. É preciso realizar estudos deste tipo com médicos e pacientes para complementar as informações coletadas.

Las guías de práctica clínica (GPC) son un “conjunto de recomendaciones basadas en una revisión sistemática de la evidencia y en la evaluación de los riesgos y beneficios de las diferentes alternativas, con el objetivo de optimizar la atención sanitaria a los pacientes” (1). Múltiples estudios han mostrado que las GPC poseen el potencial de transferir conocimiento, reducir la variabilidad clínica y mejorar la calidad de los cuidados de salud. Sin embargo, para lograr estos objetivos, las recomendaciones de las GPC deben ser factibles de implementar (2).

En Chile, en el marco de la reforma de salud, se promulgó en el año 2005 la Ley de Garantías Explícitas en Salud (GES) que garantiza el acceso, la oportunidad de atención con plazos definidos de espera, financiamiento y calidad para una serie de problemas de salud priorizados según la carga de enfermedad, la efectividad, el costo-efectividad y las preferencias sociales. Sin embargo, estos criterios de priorización no son obligatorios (3). La ley de autoridad sanitaria plantea, dentro de las funciones del Ministerio de Salud (MINSAL), establecer protocolos de atención de salud, los cuales poseen carácter referencial (4). De esta forma, el MINSAL ha definido dentro de sus funciones la elaboración de GPC para cada problema de salud GES, con la División de Prevención y Control de Enfermedades del MINSAL como la responsable de elaborar GPC bajo la asesoría metodológica del Departamento Secretaría Técnica GES y de Coordinación Evidencial y Metodológica.

Un estudio realizado en el año 2016 tuvo como objetivo evaluar la calidad metodológica de las GPC desarrolladas por la División de Prevención y Control de Enfermedades utilizando el instrumento AGREE II. Luego de aplicar este instrumento, se concluyó que las guías eran, en general, de moderada a baja calidad, con puntuaciones bajas en los dominios de rigor en la elaboración (41,4%), aplicabilidad (33,6%) y participación de los implicados (51,3%) (5).

A raíz de estos resultados, el MINSAL decidió elaborar un plan para mejorar la calidad de las GPC revisando aspectos de su desarrollo e implementación. Como primera intervención, se convocó a expertos nacionales e internacionales para elaborar un manual metodológico para el desarrollo de GPC utilizando la metodología GRADE (6, 7). Esta “guía de guías” se encuentra en la actualidad en proceso de difusión e implementación dentro y fuera de la institución, y se enfoca principalmente en describir la metodología de elaboración bajo estándares de calidad internacional, con el abordaje de estrategias generales de implementación.

La segunda intervención planificada por el equipo técnico ministerial fue analizar y optimizar la implementación de las recomendaciones contenidas en las 82 GPC de la Ley GES, elaboradas en el MINSAL. Para materializar este análisis del proceso actual de implementación se realizó el presente estudio, cuyo objetivo fue generar propuestas para optimizar el proceso de implementación de recomendaciones contenidas en GPC informadas por evidencia. Los objetivos específicos fueron: 1) definir una metodología para realizar un diagnóstico de los desafíos de implementación de las recomendaciones de las guías desarrolladas por el MINSAL, 2) aplicar dicha metodología para obtener un diagnóstico de los actuales desafíos de implementación, y 3) proponer estrategias para enfrentar dichos desafíos a distintos niveles del sistema de salud.

## MATERIALES Y MÉTODOS

Se utilizó metodología cualitativa, que se encuentra dentro de la línea de la investigación-acción y cuyo objetivo es intervenir la realidad definida como problemática, en un proceso paralelo en el que se indaga la complejidad del fenómeno observado. En el contexto de este estudio, se entendió la implementación de recomendaciones como un proceso estratégico que incluye elementos educacionales, sociales, organizacionales, tecnológicos y financieros (8). En términos prácticos, el proceso de implementación tiene como objetivo transferir las recomendaciones contenidas en las GPC al quehacer diario de la práctica clínica.

**FIGURA 1.**
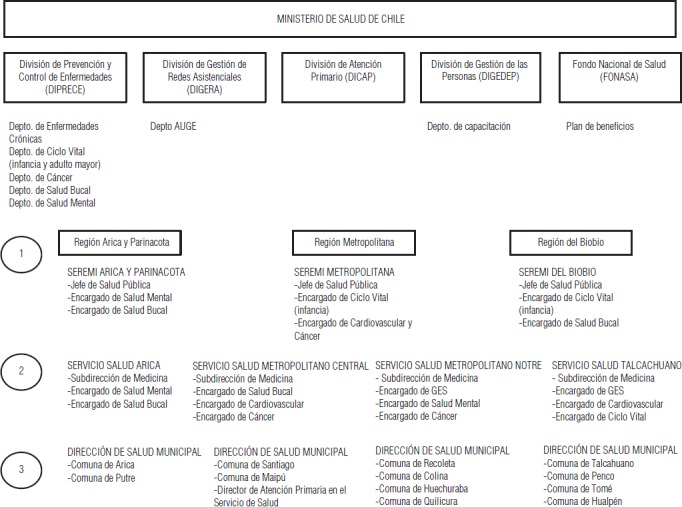
Niveles del sistema de salud chileno incluidos en este estudio según área geográfica y división institucional

Las técnicas utilizadas fueron entrevistas semiestructuradas (19 entrevistas) y grupos de discusión (nueve grupos), pues el equipo estimó fundamental indagar en profundidad el fenómeno. Se abarcaron distintos niveles del sistema público de salud chileno (figura 1), donde se incluyeron autoridades, tomadores de decisión, diseñadores de políticas públicas y funcionarios de organismos administradores de salud de distinta complejidad, quienes se seleccionaron de manera intencionada para lograr representatividad institucional y geográfica.

Este estudio recibió aprobación por parte de los Comités de Ética del Servicio de Salud Metropolitano Central, MINSAL y la Organización Panamericana de la Salud. Se diseñó un proceso de consentimiento informado estándar, por el cual cada sujeto de estudio fue informado, consultado y aceptó participar.

**FIGURA 2.**
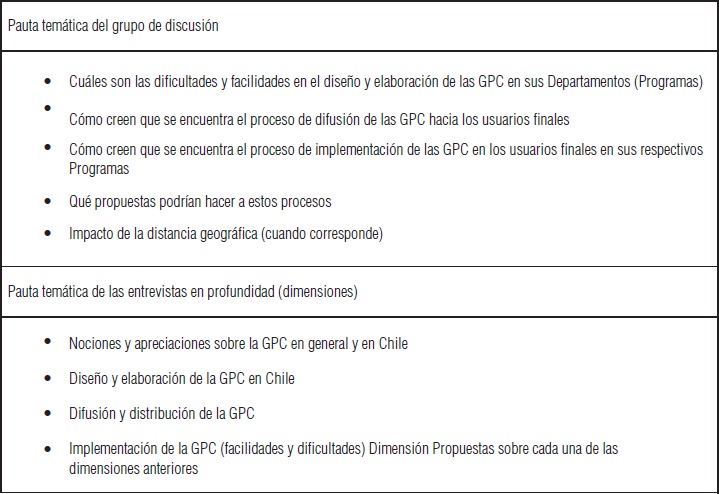
Pautas temáticas aplicadas en las entrevistas y grupos de discusión

Los instrumentos diseñados fueron organizados en torno a cuatro dimensiones preespecificadas: nociones y apreciaciones en torno a las GPC en general y en Chile, diseño y elaboración de GPC, gestión y difusión de las GPC y su implementación. El detalle del contenido de las entrevistas, grupos de discusión y dimensiones abordadas se describen en la figura 2. La aplicación en terreno de los instrumentos fue realizada por colaboradores (antropólogos y sociólogos), quienes tuvieron una inducción sobre el tema de estudio y fueron familiarizados con los instrumentos.

*GPC, guías de práctica clínica.*

Se realizaron diferentes resguardos éticos durante el desarrollo de este estudio: los investigadores en terreno fueron personas ajenas a las instituciones vinculadas al estudio y en los grupos de discusión no se reunió, en una misma instancia, a jefaturas con profesionales técnicos, con el cuidado de resguardar los equilibrios de poder entre las personas y sus funciones.

El análisis de las transcripciones de entrevistas y grupos de discusión se realizó de manera manual y mediante el *software* Atlas ti^®^, desde un marco de análisis de contenido con la categorización y la codificación de la información según las dimensiones propuestas originalmente en el diseño de los instrumentos y con la inclusión de categorías emergentes cuando fuera pertinente.

Los investigadores de la Universidad de Chile y los del MINSAL se reunieron en dos talleres en donde se analizaron los hallazgos, para así elaborar conclusiones por nivel y generales. Además, se generaron indicadores cualitativos que sirvieron de insumo para un taller de socialización de los resultados con personas clave en el proceso de elaboración e implementación de las GPC tanto a nivel público como académico. En este taller, se generaron propuestas concretas de acciones para la implementación efectiva de GPC. El trabajo conjunto entre los equipos permitió una discusión que dio pie a la implementación de los resultados del estudio por parte de los mismos actores clave. Esta experiencia permitió llevar a cabo un exhaustivo proceso de introspección institucional a nivel ministerial.

Los resultados de los talleres fueron analizados por el Departamento Secretaría Técnica GES y de Coordinación Evidencial y Metodológica para precisar los desafíos identificados, ajustarlos a un lenguaje claro y sugerir estrategias que permitirían enfrentarlos mediante una perspectiva temporal y la definición de un plan para optimizar el proceso de implementación de recomendaciones contenidas en las GPC elaboradas por el MINSAL.

## RESULTADOS

La selección de los sujetos de estudio se hizo en torno a su papel y función dentro de la institución a la que pertenecen. Las técnicas para la recolección de datos se aplicaron a nivel nacional, con muestras de las regiones de Arica y Parinacota (Norte), Biobío (Sur) y Región Metropolitana (capital), con la intención de obtener información sobre los desafíos de implementación de diferentes realidades dentro del sistema de salud. Luego se formularon conclusiones generales sobre los desafíos de implementación de recomendaciones de GPC, incluidos los hallazgos de todos los niveles estudiados.

Entre los desafíos descritos para la dimensión “nociones y apreciaciones en torno a las GPC en general y en Chile”, los participantes destacaron los múltiples significados de las guías que se manejan en los distintos niveles del sistema, lo que se traduce en diversas expectativas y usos de ellas.

En la dimensión “diseño y elaboración de GPC”, los participantes manifestaron dificultades para incorporar la variabilidad geográfica y asistencial chilena al momento de formular recomendaciones, lo cual también es percibido por el usuario final. Otro desafío es la necesidad de hacer las GPC más accesibles a pacientes y cuidadores como una forma de facilitar la implementación a nivel clínico. Como tercer elemento, los entrevistados manifestaron que la implementación de recomendaciones es un aspecto poco abordado en las GPC actuales.

En la dimensión “gestión y difusión de las GPC”, los participantes manifestaron que, en la actualidad, existen dificultades de acceso a las GPC y sus recomendaciones. Por otra parte, si bien se sugiere la necesidad de capacitar y educar a profesionales en el contenido y uso de las guías, se reconoce como desafío adicional la alta rotación del personal de salud, por lo que se deberían desarrollar estrategias de capacitación continua y una revisión permanente de las GPC por parte de los equipos de trabajo.

En la dimensión de “implementación de las GPC”, se reconoce la necesidad de reposicionar la implementación de recomendaciones como un elemento vital, ya que hasta ahora queda solo a decisión local su potencial implementación. Se reconoce que, hoy en día, no existe un proceso estandarizado que guíe el proceso de implementación ni métodos permanentes para su futura monitorización.

Entre los facilitadores que permitirían la implementación de GPC, se destaca el reconocimiento de ellas como herramienta de apoyo para la toma de decisiones en los diferentes niveles de atención. Asimismo, se reconocen los esfuerzos realizados para mejorar la calidad metodológica de las guías, basados en la estandarización de los procesos de elaboración.

**FIGURA 3.**
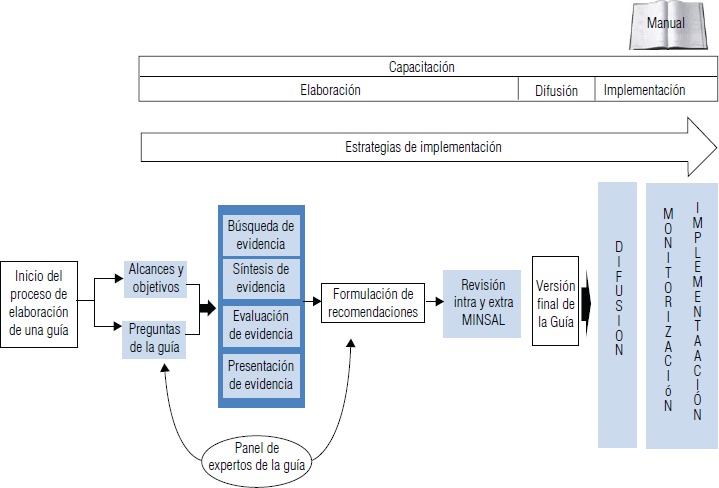
Flujograma de gestión optimizada para el desarrollo e implementación de guías de práctica clínica

A partir de estos desafíos, se elaboró un flujograma con un abordaje integrado para optimizar el proceso de implementación de GPC a lo largo de todo el curso de desarrollo de estas, el cual contiene acciones concretas a implementar para vencer las barreras identificadas (figura 3).

El flujograma se inicia con el proceso de elaboración de una guía, proponiendo una serie de acciones para cada uno de los ámbitos en el proceso de elaboración, difusión e implementación.

Respecto al “alcance y objetivos de una guía” se releva la importancia de señalar la definición de esta y el alcance según el contexto. También se destaca la necesidad de incorporar aspectos que reflejen la realidad regional (epidemiológica, gestión de red y recursos disponibles) en las preguntas de la GPC, con el fin de incluir alternativas de manejo según factibilidad de implementación, realizando un proceso de consulta pública abierta a cualquier ciudadano de las preguntas propuestas por el panel elaborador.

En el “panel elaborador de una GPC” se señala la relevancia de realizar una convocatoria pública de expertos para su participación en el panel, así como también invitar a expertos de diversas áreas geográficas que representen a distintos usuarios finales (clínicos y no clínicos). Asimismo, se indica la importancia de educar al panel elaborador sobre la definición, alcance y metodología de elaboración de las GPC.

En el ámbito de la evidencia se destaca la necesidad de que profesionales del ministerio con conocimiento de la red recojan información epidemiológica, de gestión de la red y recursos disponibles (humanos, físicos, financieros, tecnológicos) a nivel regional para cada GPC. Esta información debe ser presentada al panel elaborador de las GPC para ser considerada dentro del proceso de formulación de las recomendaciones para evaluar la factibilidad de implementación de estas. Además, se propone para esta etapa enunciar estrategias que permitan vencer las barreras de implementación identificadas para llevar a cabo cada una de las recomendaciones.

En la etapa de revisión intraministerial y externa de cada GPC se señala la necesidad de vincular las GPC con los lineamientos establecidos para la red de atención ambulatoria del país y considerar, en dicho proceso de armonización, que los documentos relacionados al tema de la GPC y dicha red sean concordantes.

Respecto a la difusión de la GPC se destaca el diseño de una página web de fácil acceso que permita informar la etapa del proceso en la que se encuentran cada una de las guías, vincularlas con documentos relacionados y ordenarlas bajo la mirada de curso de vida. Asimismo, se procura fomentar la estandarización de la estructura, formato, lenguaje y metodología para la elaboración de GPC, y preparar documentos complementarios para pacientes, cuidadores y familias considerando diferencias culturales y discapacidades de las personas así como también diferencias en el acceso a tecnología, formatos y preferencias generacionales.

Para las estrategias de implementación se propone considerar estos aspectos desde el inicio del proceso de elaboración de la GPC, así como también una consideración especial a la realidad regional (epidemiológica, gestión de red y recursos disponibles) para decidir qué recomendaciones serán implementadas y qué estrategias se utilizarán a nivel local. Además, es necesario considerar una planificación sanitaria adicional para aquellas recomendaciones que no pueden ser implementadas inmediatamente por problemas de recursos.

En capacitación en GPC se destaca la necesidad de efectuarla periódica a usuarios finales y a quienes las elaboran. Además, es necesario definir e incorporar estrategias de capacitación de temáticas de GPC para pacientes, aprovechando las potencialidades de la sociedad civil. También se indica la importancia de definir el perfil del público a educar, objetivos, metodología, frecuencia, formato y contenidos de las capacitaciones según roles, funciones y necesidades locales, y especificar los criterios de calidad que deben cumplir quienes imparten dichas capacitaciones, sobre todo cuando estas no puedan ser asumidas desde la entidad elaboradora.

**FIGURA 4.**
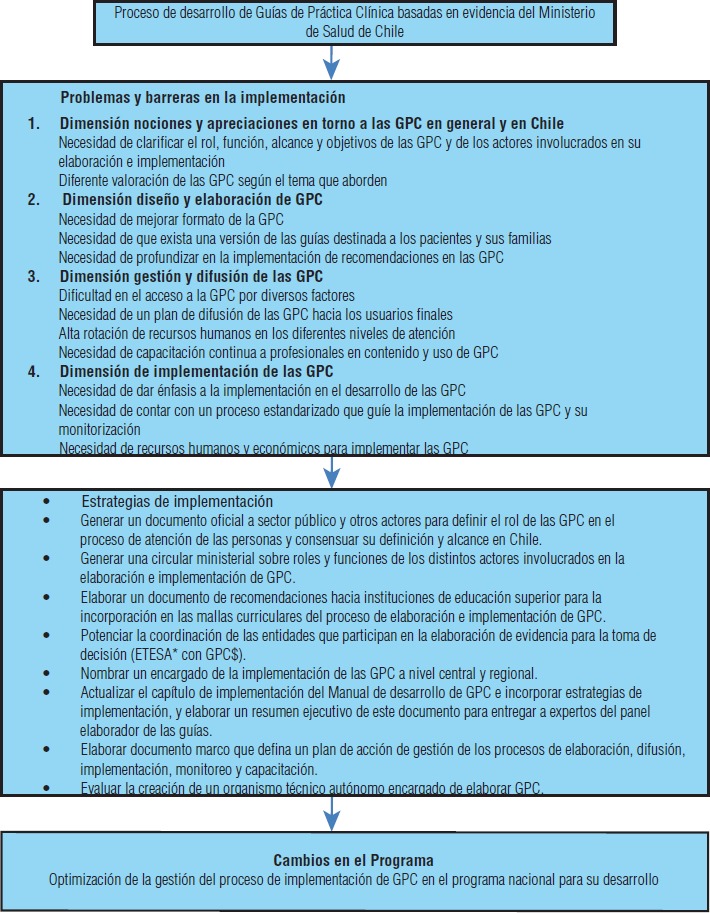
Estrategias sugeridas para enfrentar los desafíos identificados en el proceso de elaboración de GPC del Ministerio de Salud MINSAL

Se identificaron otras estrategias a implementar al interior del MINSAL para enfrentar los desafíos identificados y llevar a cabo la propuesta de flujograma con las acciones antes presentadas (figura 4). En la actualidad se trabaja en la actualización del Manual de desarrollo de GPC, en el que se consideran los aspectos antes presentados, y también en la generación de un documento marco organizacional del departamento a cargo del programa nacional para el desarrollo de GPC del MINSAL.

* Evaluación de tecnologías sanitarias

*$ Guía de práctica clínica*

## DISCUSIÓN

Las GPC y sus recomendaciones tienen como objetivo asistir a clínicos y tomadores de decisiones en la evaluación de riesgos y beneficios entre diferentes alternativas, con el fin de optimizar el cuidado de los pacientes. Si bien las guías son vistas como una forma de reducir la variabilidad clínica, lo que permite que los pacientes puedan obtener la mejor opción disponible para el cuidado de su salud, la posibilidad de que las distintas opciones o intervenciones presentadas en las recomendaciones puedan efectivamente utilizarse depende mucho del contexto. Hasta ahora, ningún estudio había profundizado en aspectos vinculados con la implementación de recomendaciones.

El MINSAL, en su misión de asistir la toma de decisiones clínicas proveyendo recomendaciones informadas por evidencia, definió una serie de intervenciones para optimizar y revisar el proceso de desarrollo de GPC. Dado que la implementación de recomendaciones solo se entiende en el contexto de la existencia de guías de alta calidad metodológica, el MINSAL ha realizado esfuerzos para asegurar esta primera condición a través de la capacitación de sus funcionarios y la elaboración de directrices técnicas para el desarrollo de GPC como el Manual Metodológico desarrollado en el año 2014, con la colaboración de instituciones externas como la Organización Panamericana de la Salud (OPS), la Universidad de McMaster en Canadá y la Pontificia Universidad Católica de Chile (6, 7). Dentro del marco de revisión y optimización de procesos es que el presente estudio corresponde al componente relacionado con los aspectos de implementación de recomendaciones.

Dentro de los hallazgos identificados, se destaca la alta valoración que existe para las GPC y la prioridad en su elaboración por parte del MINSAL, así como también las mejoras en términos de calidad metodológica que hasta la fecha se han experimentado debido a la estandarización del proceso de elaboración.

Por otra parte, se identificaron desafíos como la falta de claridad sobre el rol y alcance clínico y legal que una GPC tendría en el sistema de salud chileno, como también la falta de difusión masiva y estratégica de las GPC MINSAL. Si bien, a los efectos prácticos, la difusión de las GPC se distingue de su implementación, ambos procesos están interconectados. La literatura sugiere que no es suficiente con que las guías se difundan ampliamente y los médicos clínicos estén informados de su contenido para asegurar la adherencia e implementación de recomendaciones (9).

Otro punto a destacar es la necesidad de reconocer ciertos desafíos inherentes al sistema de salud considerando particularidades del país. Por ejemplo, la gran variabilidad geográfica, cultural, y de disponibilidad de recursos identificada por los participantes hace esperable que la “implementabilidad” de una recomendación pueda variar de un escenario a otro. Sin embargo, es importante entender que esta variabilidad debe estar reflejada en la fuerza de la recomendación y, por lo tanto, quienes formulen las recomendaciones, deben considerar su implementabilidad. Por ejemplo, una recomendación puede ser débil o condicional por un período de tiempo determinado, hasta que los recursos humanos y materiales, acceso o complementariedad con la medicina de pueblos originarios sean dilucidados y organizados en un proceso activo de implementación (10). Se sugirió, por lo tanto, que las guías ministeriales deben intentar capturar e informar a los clínicos y otros interesados sobre este tipo de desafíos y proveer alternativas de manejo cuando las condiciones de implementabilidad aún no estén dadas.

Un tercer punto fundamental que surgió es la identificación de la implementación y monitorización de recomendaciones como un proceso más en el desarrollo de las GPC, y como tal, requiere de una metodología explícita y un plan de trabajo organizado, con funciones y roles claramente definidos dentro de las diversas instituciones de salud. En este sentido, sería el propio MINSAL quien plantee estos roles y funciones y los haga llegar a las estructuras institucionales y políticas correspondientes. Junto a la definición de las estrategias mencionadas en la figura 4, se determinó también qué agentes del MINSAL debieran hacerse cargo de dichas acciones a lo largo del proceso de implementación con el fin de optimizarlo y fortalecerlo.

Un cuarto punto destaca la necesidad de capacitación continua en ámbitos metodológicos como búsqueda de evidencia, evaluación de calidad y síntesis de evidencia de los distintos actores involucrados en el proceso de elaboración de las GPC, ante lo cual es importante relevar lo que MINSAL ha realizado al respecto, con actividades de capacitación intraministerial para los elaboradores de guías.

Un último objetivo de implementación en pos de mejorar el proceso de desarrollo de GPC es la incorporación de las acciones propuestas y un resumen de los desafíos específicos de implementación en Chile en la próxima actualización de contenidos del manual metodológico para el desarrollo de guías clínicas MINSAL y en la agenda anual de capacitaciones del Ministerio.

Si bien los resultados de este estudio son particularmente relevantes para la realidad local del sistema de salud chileno, muchos de los hallazgos aquí presentados ya han sido descritos en la escasa literatura científica dedicada a su implementación (11–15). Una revisión sistemática cuyo objetivo fue sintetizar los estudios e informó sobre la efectividad de distintas intervenciones de implementación, concluyó que no existe una única estrategia clara a implementar, y que en realidad el efecto de cada una de ellas es pequeño (16). Estos hallazgos apoyan la idea de que todas las estrategias planteadas por el equipo de trabajo deben ser implementadas en conjunto para lograr el efecto esperado.

Entre las fortalezas de este estudio, podemos mencionar la metodología de investigación-acción utilizada, con la misma institución como mandante e investigada. Esto permitió posicionar la temática de estudio como un problema a abordar, al relevar el tema de las GPC dentro de la discusión de diversos acto-res que tienen influencia en la toma de decisiones en salud. Hasta este momento, esta experiencia de revisión de procesos internos ministeriales vinculados a la implementación de GPC a través de una metodología de investigación-acción es inédito en América Latina. Sin embargo, este estudio también tiene ciertas limitaciones. Por ejemplo, en esta oportunidad, no se contó con la participación de los usuarios finales de las guías, ya sean estos los clínicos o los pacientes objetivo. La información recolectada respecto de estos actores provino de otros informantes clave, quienes expusieron su propia visión de cómo los pacientes y clínicos percibían los desafíos que enfrenta el MINSAL en materia de implementación. Por otra parte, la metodología fue desarrollada sin conocer si las guías son o no aplicadas a nivel local, por lo que en muchos casos podría no ser necesario implementar las estrategias recomendadas en este estudio, ya que no cambiarán los resultados en los pacientes. En un grupo de discusión se ajustó la metodología de entrevista debido a la inasistencia de algunos actores claves citados, por lo que en última instancia se realizó una entrevista grupal.

Las posibilidades de integración y sustentabilidad de las estrategias propuestas en el proceso de implementación de las GPC son necesarias para generar un impacto en la red asistencial; por lo tanto, es importante destacar que solo podrán ser llevadas a cabo con el apoyo por parte de autoridades ministeriales, con los recursos y el tiempo necesarios para ello.

Futuras investigaciones a nivel local deben abordar problemas más específicos respecto de la implementación de recomendaciones de GPC mediante la utilización, por ejemplo, de estudios de casos o midiendo adherencia a las recomendaciones ya formuladas a modo de identificar experiencias exitosas y deficientes del proceso de implementación.

## Conclusión

Con el escenario actual como punto de partida, sumado a los esfuerzos realizados inicialmente por el MINSAL para asegurar la calidad metodológica de sus guías clínicas, es que surge ahora la necesidad de articular un plan de implementación de recomendaciones para orientar y monitorizar dicho proceso. Autoridades y otros agentes ministeriales aportaron estrategias para enfrentar dichos desafíos de obtener GPC con un alto grado de implementabilidad, considerando particularidades geográficas, culturales, y complejidad del sistema de salud chileno. Esta metodología de investigación-acción también puede ser utilizada por otros ministerios de la región interesados en intervenir sus programas de GPC.

## Declaración.

Las opiniones expresadas en este manuscrito son responsabilidad del autor y no reflejan necesariamente los criterios ni la política de la *RPSP/PAJPH* y/o de la OPS.
